# Implementing laboratory quality management systems in Ghana: A quality audit of accreditation readiness in clinical and public health laboratories (2021–2023)

**DOI:** 10.4102/ajlm.v15i1.3049

**Published:** 2026-08-27

**Authors:** Emma E. Kploanyi, David W. Dowdy, Joseph Kenu, Benjamin Buade, Benedicta Owusu-Arthur, David A. Opare, Franklin Asiedu-Bekoe, Alfred E. Yawson, Ernest Kenu, Lee F. Schroeder

**Affiliations:** 1 Department of Epidemiology and Disease Control, School of Public Health, University of Ghana, Accra, Ghana; 2 Department of Epidemiology, Bloomberg School of Public Health, Johns Hopkins University, Baltimore, Maryland, United States; 3 National Public Health and Reference Laboratory, Ghana Health Service, Accra, Ghana; 4 Division of Public Health, Ghana Health Service, Accra, Ghana; 5 Department of Community Health, College of Health Sciences, University of Ghana, Accra, Ghana; 6 Department of Pathology and Clinical Laboratories, University of Michigan, Ann Arbor, Michigan, United States

**Keywords:** laboratory quality, lower-tier facilities, diagnostic accuracy, laboratory medicine, Africa

## Abstract

**Background:**

The Stepwise Laboratory Improvement Process Towards Accreditation (SLIPTA) checklist has demonstrated improvement in laboratory quality in Africa. In Ghana, however, implementation has targeted mainly higher-tier clinical laboratories. A balanced approach across the laboratory network is needed to strengthen national laboratory quality.

**Objective:**

To evaluate the quality management performance and accreditation readiness of clinical and public health laboratories (PHLs) in Ghana.

**Methods:**

Using a modified SLIPTA checklist, we audited randomly selected clinical and PHLs from districts in the three ecological zones from February 2021 to November 2023. Analyses employed Wilcoxon rank-sum tests, Kruskal-Wallis tests, and quantile regression to assess associations between laboratory characteristics and SLIPTA scores.

**Results:**

Forty-five health facilities and four PHLs were audited: 80% of facilities were in the public sector. Laboratories performed a median of 24 (Interquartile range [IQR]: 10–70) tests daily. The median SLIPTA score was 50% (IQR: 35% – 58%), with hospitals recording the highest scores [median, IQR: 58% (51% – 61%)]. Of the 12 SLIPTA quality systems essentials, laboratories performed highest in organisation and personnel. In multivariable regression, facility location in the Northern zone (adjusted coefficient = –23, 95%CI = –38 to –8; *p* < 0.001) was negatively associated with SLIPTA scores compared to the Southern zone.

**Conclusion:**

More hospitals and PHLs had achieved a minimum one-star rating towards International Organization for Standardization accreditation readiness during the quality audit. Persistent gaps in client management, internal audits, and corrective actions require targeted training and mentoring to support effective implementation of improvement initiatives.

**What this study adds:**

This study provides the first nationwide quality audit of a representative sample of clinical laboratories and PHLs in Ghana with a balanced representation of health facilities in the tiered laboratory network, underscoring the need for a more inclusive, sustainable approach to laboratory quality improvement across the tiered network of the country.

## Introduction

The current state of medical laboratory quality is characterised by continuous efforts to enhance accuracy, safety, and efficiency in testing processes. Despite technological advancements and the implementation of quality management systems, challenges persist like variability in testing procedures, human errors, and resource limitations. The implementation of laboratory quality management systems and programmes like Strengthening Laboratory Management Towards Accreditation (SLMTA) and the Stepwise Laboratory Improvement Process Towards Accreditation (SLIPTA) has been instrumental in improving laboratory quality, serving as a cornerstone of laboratory accreditation, particularly in low-resource settings.^1,2^ Over 600 medical laboratories in sub-Saharan Africa that enrolled in these programmes became International Organization for Standardization (ISO) 15189-accredited by 2020, representing a 75% increase in accredited laboratories since 2013. However, most of these accredited laboratories were concentrated in South Africa and Kenya, where national accreditation bodies exist, with only 38 accredited laboratories in West Africa.^3,4^

In West Africa, several countries have made significant progress in implementing SLMTA and SLIPTA, with notable successes in countries such as Benin, Burkina Faso, Nigeria, Liberia, and Ghana.^5,6,7,8,9,10,11,12^ These programmes involved baseline audits and/or training, mentorship, and continuous monitoring to address gaps in quality management. Despite this, barriers to quality improvement include inadequate infrastructure, such as unreliable electricity and a lack of access to quality control materials, as well as insufficient trained personnel, limited knowledge of quality management systems, and inadequate funding for improvement projects.^5,6,13^

After Ghana adopted the SLMTA programme in 2009, three cohorts of medical laboratories were enrolled from 2011 to 2013, totalling 15 laboratories selected from the then 10 regions of Ghana. Compared to baseline SLIPTA audits with scores ranging from 13% to 42%, three laboratories achieved a minimum of one star rating (score range of 55% – 64%) at the exit audit, and approximately 88% of the laboratories that conducted follow-up audits also attained at least one star rating.^8^ Regarding laboratory performance in the quality system essentials (QSEs) during the exit audit, laboratories demonstrated high compliance in client management and customer service, as well as in process control and internal and external quality assessment. However, the performance was poor in occurrence management and internal audit. While the QSEs with high compliance varied in subsequent audits, areas for improvement persisted, including occurrence management, corrective action, and internal audit.^10,14^ This highlights the need for continuous monitoring to ascertain whether progress has been made in these areas of laboratory quality in the country towards accreditation readiness.

Laboratory accreditation benefits the continuous improvement cycle, resulting in a significant reduction in nonconformities,^15^ essential for accurate diagnoses and effective treatment. Hence, the Joint External Evaluation 2017 report for Ghana recommended that laboratory accreditation be enforced.^16^ Since the first public sector laboratory in Ghana received ISO 15189:2012 accreditation in 2017,^9^ the national reference laboratory^17^ and two regional hospital laboratories^18,19^ have also been accredited. Currently, there are only five ISO 15189 and three ISO 9001:2015-accredited clinical and public health laboratories (PHLs) in the Ghanaian public sector^17,18,20,21,22,23^ out of the over 800 clinical laboratories.^24^ International Organization for Standardization 15189 accreditation is specifically designed for medical laboratories, whereas ISO 9001 is a generic standard applied across various organisations.^25,26^

Importantly, the SLIPTA audits in the Ghanaian public health sector have primarily involved higher-tier clinical laboratories, with a few district hospital laboratories included, which is not a representative sampling of facility tiers in Ghana’s health system.^8,10,14^ However, the guidance for SLMTA implementation recommends that countries prioritise the tiered laboratory network when selecting laboratories for programmes as it ensures a strategic and phased approach to improving laboratory quality.^27^ Hence, this study conducted a quality audit of representatively sampled clinical laboratories and PHLs from the national to sub-district level across the three ecological zones of Ghana.

## Methods

### Ethical considerations

The Ghana Health Service Ethical Review Committee (reference number: GHS-ERC 005/05/19) and the Noguchi Memorial Institute for Medical Research Institutional Review Board (reference number: NMIMR-IRB CPN 069/18-19) provided ethical approval for the study. Moreover, prior authorisation was secured from the Director-General of the GHS before the study commenced. Written informed consent was acquired from each participant before they were interviewed.

### Study design

A baseline quality audit was conducted using a cross-sectional survey design from 23 February 2021 to 22 November 2023, in laboratories across Ghana.

### Study setting

The study assessed clinical laboratories from different tiers, including hospitals, polyclinics, health centres, and Community-based Health Planning and Services (CHPS) facilities in Ghana’s public, private, and mission sectors. All four PHLs in the country were also included.

### Sampling

The study employed a multi-stage cluster sampling method using a list of all healthcare facilities from the public, private, and mission/religious sectors available in the national District Health Information Management System 2. To account for the different facility tiers and geographic locations, Ghana was first stratified into its three ecological zones: Northern, Middle, and Southern. Then, the population density in each district, whether above or below the median, was combined with the distance from the highest-tier health facility in each of the 261 districts to the nearest public health laboratory (national or zonal). Twelve districts in Ghana were selected randomly at this stage of sampling, requiring that no two districts come from the same region (there are 16 regions in Ghana).

The facilities were categorised into ascending tier-groups: CHPS, health centres, and three hospital tier-groups (polyclinics, hospitals, and district hospitals). A total of 48 facilities were selected, randomly four from each district, to include two hospitals (prioritising the highest-tier groups present), a health centre, and a CHPS facility. In the case where only one hospital was available, two health centres were selected. In addition, all four PHLs in the country were surveyed.

### Data collection

#### Design of the survey tool

The study adapted a modified World Health Organization Regional Office for Africa SLIPTA checklist, which was utilised by Elbireer et al.^28^ in Uganda. This modified tool retained the 12 sections corresponding to the 12 QSEs and comprised 81 checklist questions, totalling 179 points. An electronic version of the checklist hosted on Research Electronic Data Capture was administered.

#### Administration of the modified Stepwise Laboratory Improvement Process Towards Accreditation checklist

Research assistants used for data collection were trained by a laboratory quality manager and certified ISO assessor to equip them with the requisite laboratory quality assessment skills, such as probing, observation, and judgement, to ensure data reliability.^29^ Three training sessions were conducted before data collection. The tool was piloted at two facilities: a district hospital and a regional hospital to calibrate the interpretation of the modified SLIPTA checklist by the assessors. During this exercise, research assistants compared and reconciled scoring decisions under the guidance of the certified quality manager, ensuring consistent application of checklist criteria across all subsequent audits. The audits relied on multiple verification methods, including document review, direct observation, staff interviews, and triangulation of evidence, to validate reported practices.

### Data management and statistical analysis

The survey data from Research Electronic Data Capture were cleaned and analysed using STATA version 18, StataCorp LLC, College Station, Texas, USA. The outcome variable, the total percentage SLIPTA score, was calculated as the sum of all scores from the modified SLIPTA checklist, divided by the total score of 179, and then multiplied by 100. A similar calculation was applied separately for each of the 12 sections using the total scores for each section (Table 1).

**TABLE 1:** Scoring template used for the modified Stepwise Laboratory Improvement Process Towards Accreditation checklist administered during the baseline quality audit, Ghana, February 2021 to November 2023.

Section	Total score
1: Documents & records (7 items)	18
2: Management reviews (3 items)	6
3: Organisation & personnel (5 items)	14
4: Client management (1 item)	3
5: Equipment (8 items)	17
6: Internal audit (1 item)	3
7: Purchasing & inventory (8 items)	16
8: Information management (6 items)	12
9: Process control, internal & external quality assessment (EQA) (14 items)	30
10: Corrective actions (4 items)	10
11: Occurrence/Incident management & process improvement (3 items)	7
12: Facilities and biosafety (21 items)	42

**Total**	**179**

For quantitative variables: percentage SLIPTA score; testing volume defined as the average volume of tests performed each day per laboratory; the number of personnel by different cadre (including laboratory technologists or scientists, laboratory technicians, laboratory assistants or microscopists and other healthcare staff); the median and interquartile range, as well as mean and standard deviation were reported. Categorical variables included facility type (public sector, private or mission or religious); facility tier (CHPS, health centre or clinic, polyclinic, hospital (merging hospitals and district hospitals, or public health laboratory); laboratory complexity (point-of-care [POC] testing or more complex processes labelled as moderate to high complexity); and presence or absence of temperature-sensitive equipment in the laboratory. These variables were reported using frequencies and proportions. The descriptive statistics were summarised in tables and graphs.

The ordinary least squares regression model was used to assess the relationship between the average number of tests performed daily by laboratories and the average number of tests performed per laboratory personnel daily. Regression diagnostics were performed, including the Breusch-Pagan/Cook-Weisberg test for heteroscedasticity and Cook’s distance for influential observations.

Univariable analysis was conducted using the Wilcoxon rank-sum test to examine the association between SLIPTA scores and binary variables, while the Kruskal-Wallis test was employed to evaluate the association between SLIPTA scores and variables with more than two categories. Testing for multicollinearity (Table S1) was performed using Fisher’s exact test at a significance level set at *p* < 0.05 to select variables for quantile regression multivariable analysis. Laboratory complexity was significantly associated with all other variables except the ecological zone; hence, only these two were included in the multivariable analysis.

## Results

The SLIPTA audit was conducted at 45 health facilities and four PHLs across the country. The approximate time required to administer the checklist varied from 30 min to 2 h, depending on the facility type: ~30 min at CHPS facilities, ~1 h at health centres/clinics, and up to ~ 2 h at polyclinics and hospitals.

The audited facilities were equally distributed between the ecological zones, except for three facilities that could not be audited in the Southern zone. Most (80%) of the health facilities were under the public sector; of the five facility tiers, most of the audits were carried out in health centres/clinics (35%), whereas polyclinics (8%) were the least represented. Approximately 55% of the laboratories had moderate to high complexity, 67% used temperature-sensitive equipment, and 59% reported having laboratory scientists working in their facilities (Table 2).

**TABLE 2:** The distribution of percentage Stepwise Laboratory Improvement Process Towards Accreditation scores by characteristics of laboratories assessed, Ghana, February 2021 to November 2023 (*N* = 49).

Laboratory characteristics	*n*	%	SLIPTA scores (%)		*p*
			Median	Interquartile range (LQ–UQ)	
**Ecological zone**	-	-	-		< 0.001*
Northern	17	35	34	13–50	-
Middle	17	35	47	39–56	-
Southern	15	30	60	54–61	-
**Laboratory type**	-	-	-	-	0.040*
Public	39	80	44	28–56	-
Private	7	14	59	54–61	-
Mission/Religious institution	3	6	57	13–59	-
**Facility tier**	-	-	-	-	0.015*
CHPS	11	22	35	12–44	-
Health Centre/Clinic	17	35	42	24–56	-
Polyclinic	4	8	49	41–56	-
Hospital	13	27	58	51–61	-
PHL	4	8	57	53–59	-
**Complexity of laboratory**	-	-	-	-	< 0.001*
Point of care	22	45	31	13–44	-
Moderate/High complexity	27	55	56	48–61	-
**Temperature-sensitive equipment**	-	-	-	-	< 0.001*
No	16	33	35	20–46	-
Yes	33	67	55	42–61	-
**Laboratory scientist in facility**	-	-	-	-	0.041*
No	29	59	42	19–56	-
Yes	20	41	54	46–59	-

SLIPTA, Stepwise Laboratory Improvement Process Towards Accreditation; LQ, Lower Quartile; UQ, Upper Quartile; CHPS: Community-based Health Planning and Services.

* Statistical significance set at *p* < 0.05.

**FIGURE 1:**
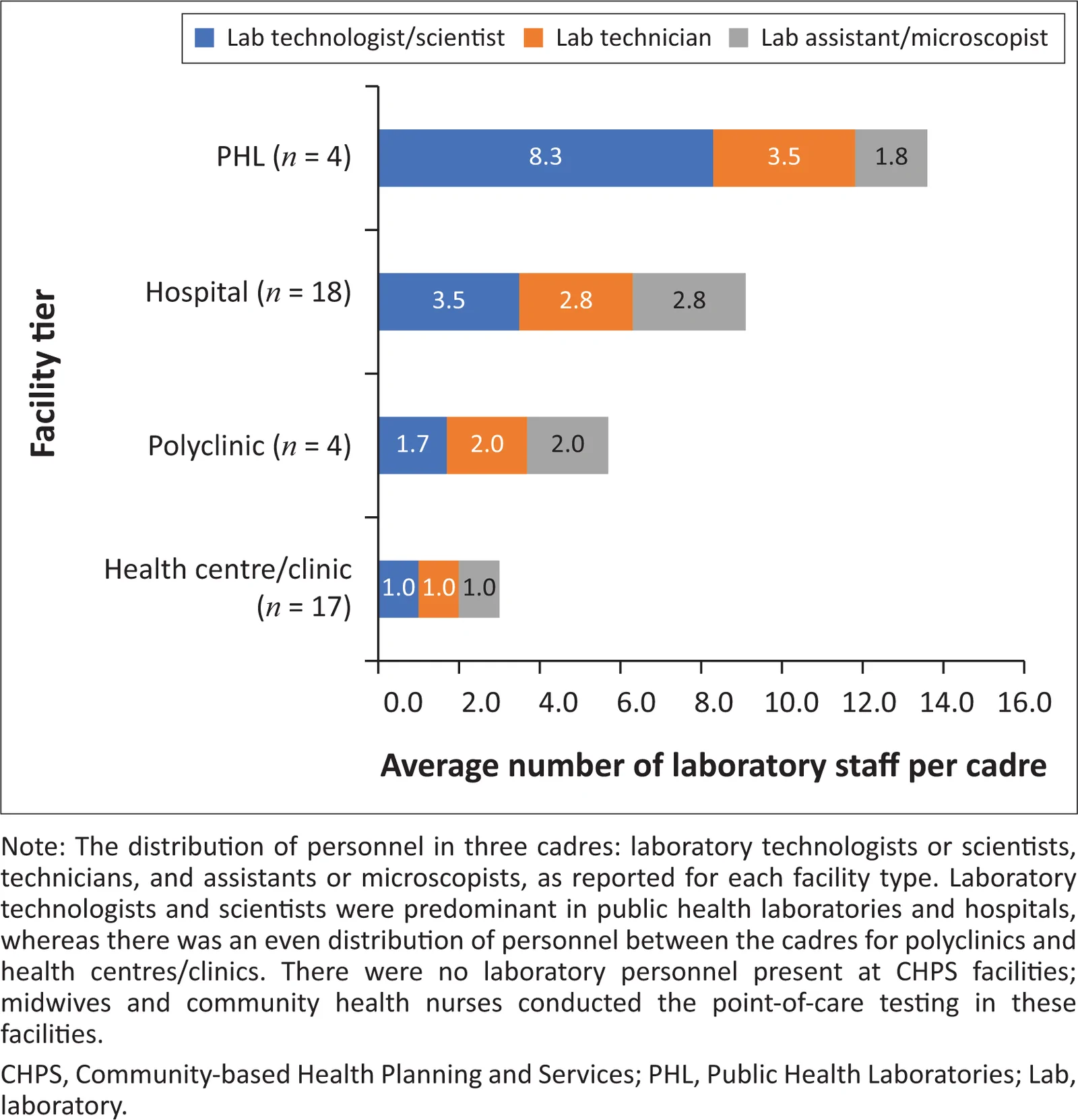
Laboratory staff cadre by facility type with moderate to high laboratory complexity, Ghana, February 2021 to November 2023.

Of the five facility tiers audited, only CHPS facilities did not have any laboratory personnel, whether laboratory technologists/scientists, technicians, or assistants/microscopists. Midwives and community health nurses conducted the POC testing at CHPS facilities. The PHLs recorded the highest number of laboratory staff per facility: an average of 8.3 laboratory technologists/scientists, 3.5 technicians, and 1.8 assistants/microscopists (Figure 1).

**FIGURE 2:**
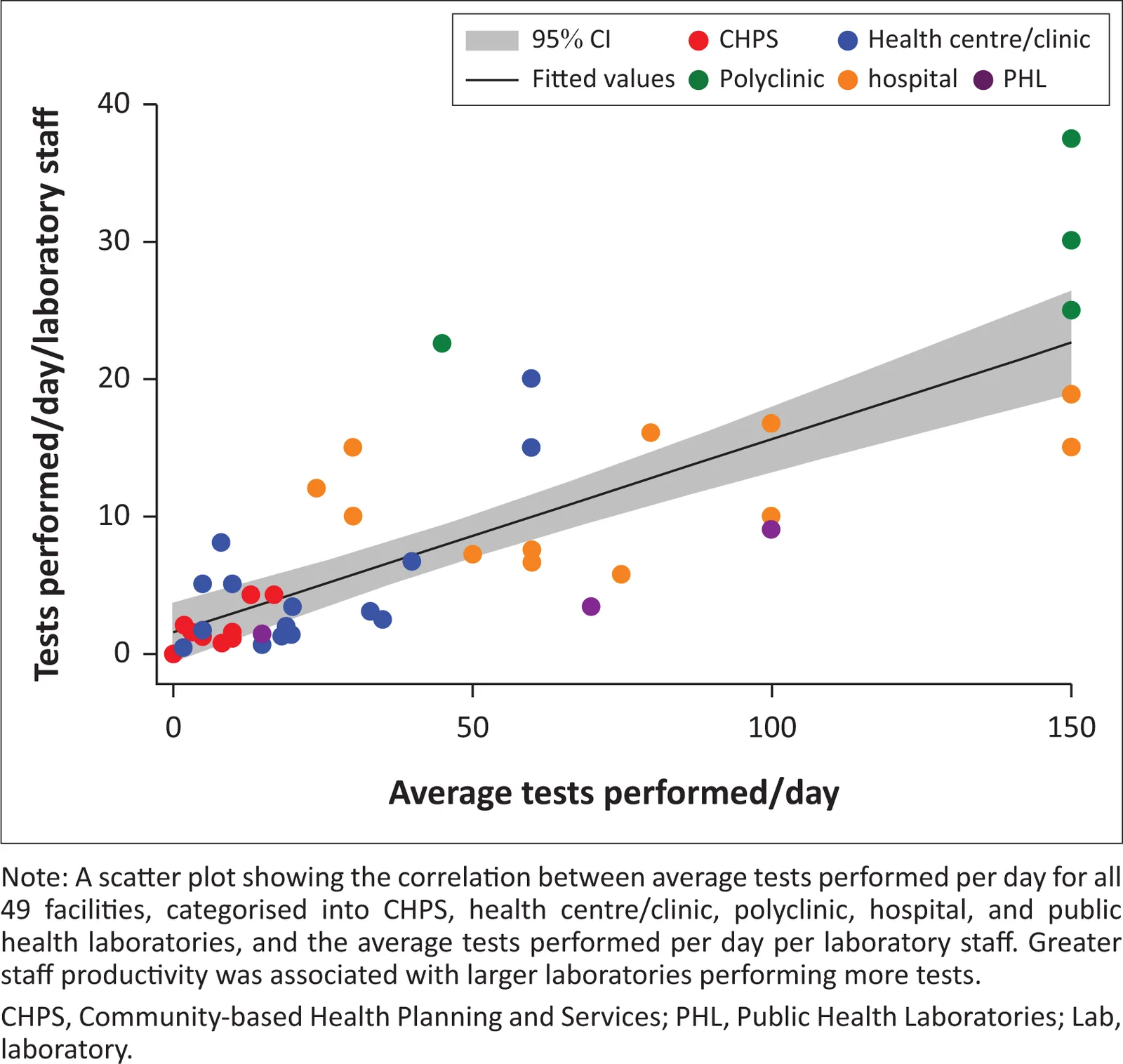
Correlation between the average tests performed per day in a facility and the mean number of tests performed per laboratory staff per day, Ghana, February 2021 to November 2023.

Health centres/clinics reported an average of one laboratory personnel for each cadre. A median of 24 (interquartile range [IQR]: 10–70) tests were performed daily in the laboratories, with each laboratory personnel performing a median of 5 (IQR: 1.5 – 10) tests daily (Figure 2). Influential data points identified by the Cook’s *D* test (Cook’s *D* > 4/*n*) were present, but removal did not influence the coefficient or *p*-value of the regression meaningfully (ordinary least squares with influential observations: intercept = 1.54, slope = 0.14; *p* < 0.001 and ordinary least squares without influential observations: intercept = 1.84, slope = 0.12; *p* < 0.001); thus, these data points were included in the analysis. However, ordinary least squares’ regression violated the assumption of homoskedasticity (*p* < 0.001); therefore, the *M*-estimation approach for robust regression was employed. This yielded a slope of 0.10 and intercept of 1.40 (*p* < 0.001).

**FIGURE 3:**
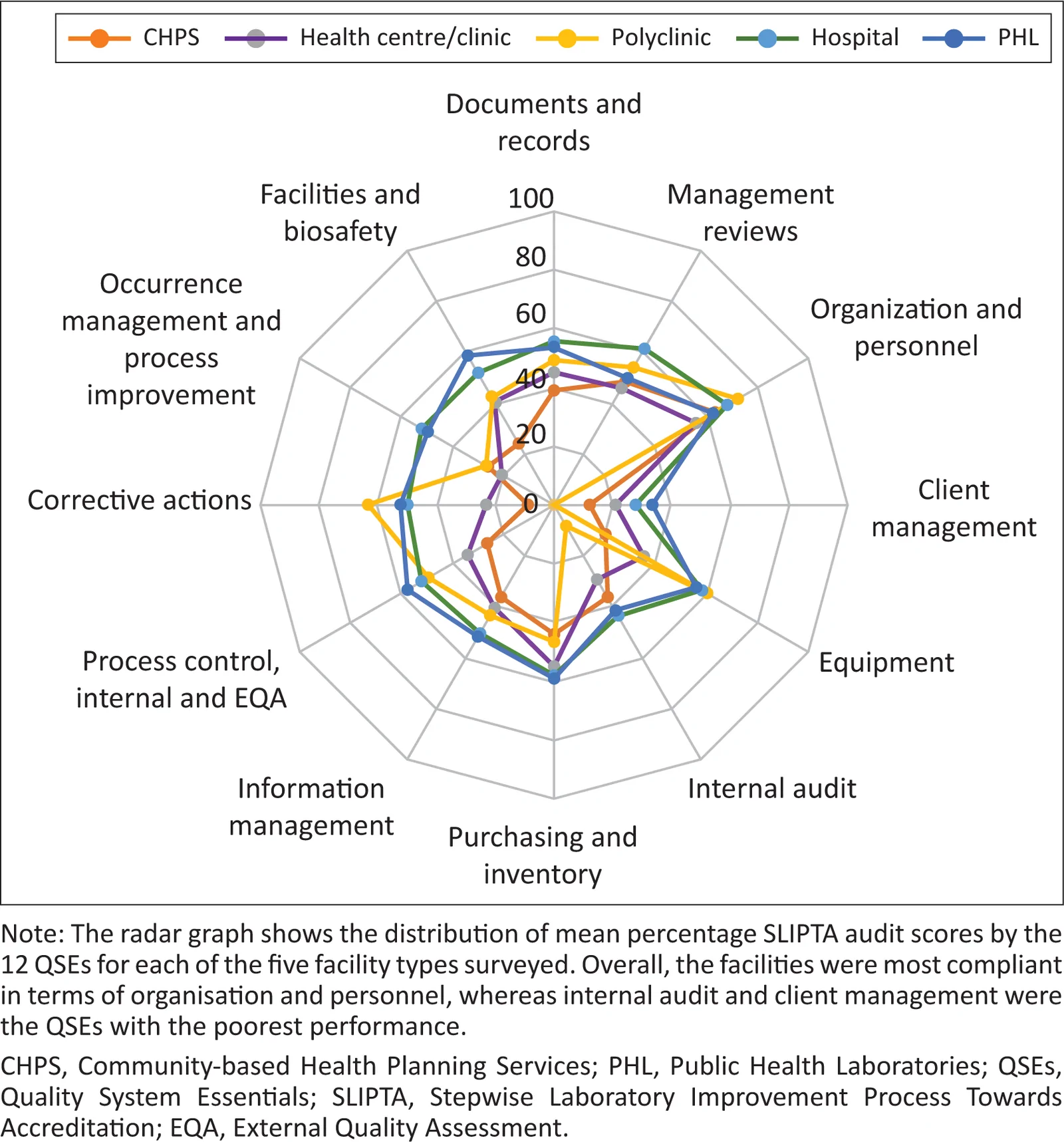
Compliance rate of the 12 Quality System Essentials assessed in 49 laboratories, Ghana, February 2021 to November 2023.

The median percentage SLIPTA audit score for all facilities was 50% (IQR: 35% – 58%). Overall, hospitals recorded the highest audit scores (Median [IQR]: 58% [51% – 61%]), whereas CHPS facilities recorded the least (Median [IQR]: 35% [12% – 44%]) (Table 2). Of the 12 SLIPTA audit sections, organisation and personnel performed the highest, whereas client management was the poorest. Specifically, polyclinic and hospital laboratories performed highest in organisation and personnel, and equipment; PHLs, hospitals, and health centres in purchasing and inventory; and hospitals in management reviews, and polyclinics in corrective action. Aside from client management, polyclinics also performed poorly in internal audit, whereas CHPS and health centres/clinics performed poorly in corrective action (Figure 3).

All laboratory characteristics assessed were significantly associated with the percentage SLIPTA audit scores estimated in the univariate analysis (Table 2). After addressing multicollinearity (Online Supplementary Table 1), multivariable regression found the conditional median percentage SLIPTA score for the Northern ecological zone was 23 points (95%CI [adjusted coefficient: aCoef] –38 to –8; *p* < 0.001), lower than that of the Southern ecological zone (Table 3).

**TABLE 3:** Quantile regression analysis of laboratory quality using percentage Stepwise Laboratory Improvement Process Towards Accreditation scores, Ghana, February 2021 to November 2023.

Laboratory characteristics	uCoef	95%CI	*p*	aCoef	95%CI	*p*
**Ecological Zone**	-	-	0.007*	-	-	0.001*
Southern	Ref	Ref	-	Ref	Ref	-
Northern	−25.42	−40.71, −10.12	-	−22.91	−37.90, −7.91	-
Middle	−12.57	−27.86, −2.72	-	−6.98	−21.48, 7.51	-
**Facility tier**	-	-	0.175	-	-	0.138
Hospital	Ref	Ref	-	Ref	Ref	-
CHPS	−23.18	−43.13, −3.24	-	−18.72	−35.13, −2.30	-
Health Centre/Clinic	−15.92	−33.86, 2.02	-	−11.45	−26.73, 3.83	-
Polyclinic	−11.17	−39.01, 16.67	-	−2.79	−26.24, 20.65	-
PHL	−2.23	−30.08, 25.61	-	3.35	−19.00, 25.71	-

Note: Testing for multicollinearity was performed using Fisher’s exact test at a significance level set at *p* < 0.05 to select variables for quantile regression multivariable analysis. Laboratory complexity was significantly associated with all other variables, except the ecological zone; therefore, only these two were included in the multivariable analysis.

uCoef, unadjusted coefficient; aCoef, adjusted coefficient; CI, confidence interval; SLIPTA, Stepwise Laboratory Improvement Process Towards Accreditation; CHPS: Community-based Health Planning and Services; Ref, reference.

* Statistical significance set at *p* < 0.05.

## Discussion

A baseline quality audit was conducted on a representative sample of clinical and PHLs across Ghana. Laboratory personnel, including laboratory technologists/scientists, technicians, and assistants/microscopists, were present in all facility types except CHPS, although they conducted a limited volume of tests on average. At least half of the hospital and PHLs assessed had attained quality compliance equivalent to one star, whereas the other facility tiers indicated lower SLIPTA scores. Of the 12 SLIPTA QSEs, laboratory compliance was highest in organisation and personnel, but lowest in client management and internal audit. A facility’s location in the Northern zone and the exclusive use of POC testing were negatively associated with SLIPTA audit scores.

The baseline compliance rates across African countries using the SLIPTA checklist vary, reflecting the diverse starting points of laboratories in their accreditation journey. The current study found a median score of 49.7% for 49 audited laboratories across Ghana. By comparison, relatively lower baseline scores have been reported in other countries over the past decade. At the initial stages of SLIPTA implementation, after the World Health Organization launched the stepwise laboratory accreditation preparedness scheme in the African region in 2009, audit scores could be as low as 18%, as reported in a regional hospital laboratory in Cameroon.^30^ In Nigeria, four out of six clinical laboratories surveyed at the time had zero stars during the baseline assessment,^11^ and in Rwanda, between 2010 and 2013, 14 out of 15 laboratories audited recorded zero stars.^31^

Most of the laboratories surveyed in the African region were in the higher-tier category, ranging from district hospital laboratories to central referral laboratories.^11,30,31,32^ Also, previous quality audits in Ghana involved public sector laboratories from the district to the national level. Baseline audit scores ranged from 5% to 55%, except for one facility with a one-star rating.^8,10,14^ This study highlights improvements in quality management over the past decade, as evidenced by half of the audited hospital and PHLs attaining quality compliance equivalent to one star. However, it is noteworthy that the previous audits compared to our audit were not conducted on a representative sample of facilities. Moreover, at the time of the survey, the National Public Health and Reference Laboratory had received ISO 15189:2022 accreditation, and the other three PHLs also had ISO 9001 accreditation. The remaining 45 laboratories were being surveyed for the first time. These findings were corroborated by the 2023 International Health Regulations (2005) State Party Self-assessment report, which assigned an 80% score for laboratory quality system^33^ compared to 40% in 2017 (a score of 2 on a scale of 1–5).^16^ This could be attributed to national laboratory standards implemented through supportive supervision, quality audits, and the mandatory licensing of laboratories by the Health Facilities Regulatory Authority using basic quality requirements.^16,24,33^

Eight years after the first public sector laboratory in Ghana received ISO 15189:2012 accreditation,^9^ no district or sub-district laboratory has been enrolled in an ISO accreditation programme, indicating a skewed focus on strengthening laboratory capacity and quality-assured service delivery at higher-tier facilities in the country. However, most patients initially visit lower-tier facilities and are likely to receive diagnoses based on the results from their laboratories. The higher-tier facilities are more likely to intervene during referrals. The fact that some hospital laboratories from the district and sub-district levels indicated compliance equivalent to one star in this study is promising and suggests their readiness to be enrolled in SLMTA programmes to strengthen their capacity for accreditation. This is even more important as the country scales up the implementation of the Network of Practice model, where health centres function as hubs for referral of cases within the sub-district.^34^

The audited facilities exhibited high compliance in organisation and personnel, indicating that staffing and organisational structures are relatively well-established in these laboratories. Similar findings were reported in the early stages of administering the SLIPTA checklist in Ghana.^8,10^ Our survey found medical laboratory professionals, including laboratory scientists, technologists, technicians, assistants, and microscopists, available at all facility levels, except for CHPS facilities, where midwives and community health nurses conducted POC testing. In line with the country’s staffing norms for clinical personnel, hospitals and PHLs reported a higher number of biomedical scientists and technologists. The number of personnel in this cadre met the minimum requirement for each facility tier. However, the number of laboratory technicians and assistants fell short of these norms, except for the health centres that met the minimum requirements.^35^ An earlier review of health workforce requirements estimated a staff availability gap of 85% for laboratory technicians and about 30% for biomedical scientists.^4^ In our study, such large laboratory personnel gaps were not found although test volumes per worker were relatively low. Thus, test volumes were most likely limited by instrumentation and lack of samples rather than by staffing constraints. This is supported by the observed productivity gains per worker in higher-tier laboratories in our study, presumably because of larger patient demand and utilisation of automated analysers.

The situation regarding the poorly performing QSEs remains similar to that in a prior study.^10^ Client management, internal audits, and corrective actions were the QSEs’ least compliant, highlighting the limited focus on client satisfaction and feedback mechanisms, as well as the inadequate capacity to conduct regular internal audits and to implement corrective actions.^8,10^ The latter have been reported as facilitators of improvement in other QSEs, as they form part of the improvement management stage,^36^ which is crucial for sustaining the continuous improvement cycle.

Although recommendations from previous audits emphasised the need to incorporate training on internal audit, streamline administrative tasks, and enhance communication channels,^8,10^ evidence-based training and tailored mentoring to implement improvement projects related to these areas need to be intensified to achieve the desired impact. A survey of training needs among medical laboratory professionals in Ghana identified quality management systems as the highest-ranking topic for training based on domains for continuing professional development training.^37^ Evidently, laboratory professionals have identified inadequate training as a key challenge,^38^ which could be a contributing factor to the noncompliance with quality standards identified by this study, as we did not find staff workload to be a barrier to quality testing.

Facilities in the Northern zone recorded lower audit scores compared to the other two zones, thus predicting the least readiness for accreditation. An earlier survey of healthcare facilities in this zone revealed a lack of resources, including laboratory equipment, supplies, and even a stable power supply. Of the 72 tests assessed on the World Health Organization Essential Diagnostics List, the laboratories could only test for 36 of them.^38^ The five northern regions, although populous, are disproportionately affected by poverty, with the Ghana Statistical Service reporting multidimensional poverty rates above the national average of 41.3% for all of them as of the end of 2023.^39^ While the government must prioritise the Northern zone in developing the necessary laboratory capacity for quality services, other innovative ways of building capacity could also be explored. As the country prepares to scale up the Network of Practice model to all regions, efforts should be invested in strengthening public–private partnerships to pool resources and expertise.^40^ In addition, facilities could leverage digital health technologies, such as an online marketplace for specimen transport to higher-tier facilities in the network,^41^ which has been piloted previously in this setting. The health directorates and related organised groups in these regions could collaborate to crowd-fund quality management system improvement projects. Crowdfunding has been proven to be an effective means of raising funds for healthcare delivery.^42^

### Limitations

Our study has a few limitations. We administered a modified SLIPTA checklist, which has been used in other countries but differed from the complete checklist previously performed in Ghana.^8^ Also, the SLIPTA checklist is designed for administration in laboratories, not for POC testing, which is considered ‘waived’ from many regulatory requirements. Hence, lower SLIPTA scores in POC testing facilities, such as health centres and CHPS, should not be interpreted as negative evaluations of these facilities. If a facility only conducts waived testing, as per regulatory authorities such as the Food and Drugs Authority (and most POC tests are waived), then the requirements for some checklist items may not be necessary. In addition, the field research assistants who administered the checklist were not qualified auditors, although a certified laboratory auditor trained them. Hence, we recommend follow-up audits by qualified auditors, especially in one-star facilities using the complete SLIPTA checklist in preparation for enrolling them in SLMTA. Surveillance audits should also be conducted in already accredited laboratories.

### Conclusion

Laboratory quality management has improved over the past decade, particularly at higher-tier facilities in Ghana. In this study, more hospitals and PHLs had achieved a minimum one-star rating towards ISO accreditation readiness. However, there was a geographical disparity in quality management, as facilities in the Northern zone indicated the least readiness for ISO accreditation. Hence, efforts to expand laboratory capacity for quality management should prioritise facilities in the Northern zone and lower-tier facilities as the country scales up the implementation of the Network of Practice model. Facilities exhibited high compliance in organisation and personnel, with medical laboratory professionals available at all facility types, excluding CHPS facilities. A need exists for a root cause analysis of the least compliant quality systems essentials, including client management, internal audits, and corrective actions, to inform evidence-based training and tailored mentoring for implementing improvement projects in these areas.
